# Transradial Approach for Pediatric Diagnostic and Neurointerventional Surgery: Single‐Center Case Series and Systematic Review

**DOI:** 10.1161/SVIN.122.000411

**Published:** 2022-08-15

**Authors:** Ammad A. Baig, Jenna Neumaier, Yusuf J. Hashmi, Muhammad Waqas, Justin M. Cappuzzo, Andre Monteiro, Hamid H. Rai, Wasiq Khawar, Kenneth V. Snyder, Elad I. Levy, Adnan H Siddiqui, Renee M. Reynolds, Jason M. Davies

**Affiliations:** ^1^ Department of Neurosurgery Jacobs School of Medicine and Biomedical Sciences University at Buffalo Buffalo NY; ^2^ Department of Neurosurgery Gates Vascular Institute at Kaleida Health Buffalo NY; ^3^ Jacobs School of Medicine and Biomedical Sciences University at Buffalo Buffalo NY; ^4^ Canon Stroke and Vascular Research Center University at Buffalo Buffalo NY; ^5^ Jacobs Institute Buffalo NY; ^6^ Department of Radiology Jacobs School of Medicine and Biomedical Sciences University at Buffalo Buffalo NY; ^7^ Department of Neurosurgery John R. Oishei Children's Hospital, Kaleida Health Buffalo NY; ^8^ Department of Bioinformatics Jacobs School of Medicine and Biomedical Sciences University at Buffalo Buffalo NY

**Keywords:** angiography, case series, diagnostic, pediatric, review, stroke, transradial, transfemoral

## Abstract

**Background:**

Recent widespread adoption of a transradial approach in adults has encouraged its use and expansion in children; however, the safety and feasibility of the transradial approach in the latter has not been established. We evaluated these characteristics in our pediatric case series and compared our results with those in the literature.

**Methods:**

Our prospectively maintained database was retrospectively searched for consecutive patients ≤18 years of age who underwent diagnostic and interventional neuroangiography through the transradial approach. Patient demographics, indications for the procedure, use of ultrasound guidance, arterial size at the access site, intra‐ and postprocedure complications, and outcomes were recorded. For the literature review, systematic searches of PubMed, MEDLINE, and Embase databases were conducted using keywords with Boolean operators (“radial artery” AND “pediatric”) for studies published in English between January 2000 and September 2021. Continuous variables were reported as means or medians and respective standard deviations and interquartile ranges according to data normality. Categorical variables were reported as frequencies.

**Results:**

Twenty‐one patients were included in our series (mean age, 16.6±2.23 years, range 9–17 years; male sex, 11 [52.4%]). The transradial approach was used for diagnostic angiography in 15 cases (71.4%) and intervention in 6 (28.6%). Ultrasound guidance was used and a “radial cocktail” (verapamil‐heparin‐nitroglycerin) was given in all cases. Mean radial artery access‐site diameter was 2.2±0.46 mm. Two cases (9.5%) required conversion to femoral access. Two patients (9.5%) suffered reversible vasospasm. No radial artery occlusion or permanent neurologic deficits were recorded. The systematic review showed results similar to ours for vasospasm rates (6.3% and 9.5%, respectively) and procedural outcomes (92% and 91%, respectively). [Correction added on November 8, 2022, after first online publication: In the preceding sentence, the value 7% was changed to 6.3%, and the value 93% was changed to 92%.]

**Conclusion:**

Our results and the literature review demonstrate that the transradial approach is a safe and feasible option for pediatric patients. Routine use of ultrasound guidance, selection of appropriately sized catheters, and prophylactic use of vasodilators and antispasmodics can help ensure the success of the procedure and limit common access‐site complications.

**Nonstandard Abbreviations and Acronyms**
TFA: transfemoral approach
TRA: transradial approach

**Clinical Perspective**

**What Is New?**

- With the widespread adoption of the transradial approach for diagnostic and interventional neuroangiography, we demonstrate the expansion of this technique in the pediatric patient population.

**What Are the Clinical Implications?**

- The results of our series and systematic review indicate that the transradial approach is safe and effective for pediatric cases when routinely used in conjunction with vasodilators, antispasmodics, and ultrasound guidance.

Although pediatric neuroangiography has been practiced since the early 2000s, it has not been reported as comprehensively as adult cerebral angiography.^1^ Until recently, a blanket transfemoral approach (TFA) was adopted by the majority of neurointerventionists. This contrasts sharply with modern cardiovascular practice, wherein a transradial approach (TRA) is more commonly used because of a number of randomized controlled trials that showed clear superiority of the TRA over the TFA.^2^, ^3^, ^4^, ^5^, ^6^, ^7^ Advantages of the TRA include few access‐site complications, improved patient satisfaction and outcomes, shorter hospital length of stay, and lower overall complication rates.^2^, ^6^, ^8^, ^9^ The American Heart Association, underscoring the substantive nature of evidence, reinforces a “radial first” approach.^10^

Recently, the shift to radial approaches has also been observed in neurointerventional surgery, evidenced by a substantial increase in the number of operators performing both diagnostic and interventional procedures using the TRA and reporting technical feasibility with encouraging outcomes and low complication rates.^8^, ^9^, ^11^, ^12^, ^13^, ^14^, ^15^ The overwhelming majority of these reports include only adult patients, with just a few reporting pediatric cases.^16^, ^17^, ^18^, ^19^ Majmundar et al published the first case series describing pediatric neuroangiography using the TRA in 2018, comprising a small sample size of 4 patients ≤18 years of age.^16^ This paucity of data on the TRA for the pediatric population can be partially attributed to the relative rarity of pediatric pathologies that require neurointervention.^16^, ^18^ In addition, the perceived risk of vasospasm, occlusion, or dissection is felt to be higher.^20^ Moreover, the existence of congenital radial artery anomalies and the added difficulty of navigating through smaller‐diameter radial vasculature adds to the challenge.^20^

Recently, improved operator experience and advances in neurointervention device technology have allowed for expansion of the TRA beyond adult patients with more frequent practice in pediatric cases.^16^, ^17^, ^18^, ^19^ We aimed to report our experience with the TRA and highlight the technical and procedural details in the pediatric population. Moreover, because of the scarcity of randomized data and small number of observational studies, we intended to perform a systematic review and pooled analysis of the literature over the past decade to evaluate safety, clinical outcomes, and feasibility of TRA in pediatric cases.

## Methods

The data that support the findings of this study are available from the corresponding author on reasonable request.

### Patient Selection and Data Collection

A prospectively maintained database was retrospectively searched to identify consecutive pediatric patients who underwent diagnostic neuroangiography, neurointervention, or both at our institution. Each patient or a legally authorized representative provided informed consent for these procedures. Institutional review board approval was obtained to conduct the study (IRB No. STUDY00003451).

Keeping with the normal practice of medicine, patients ≤18 years of age were defined as pediatric. These patients were identified during the time period from January 2015 to September 2021, and only those who underwent a TRA were included. Demographic characteristics such as sex, age, height, and weight were recorded. For descriptive purposes, patient demographics and procedural characteristics were recorded separately for the diagnostic and interventional subgroups. Indications for the procedure, use of ultrasound guidance for localization of the radial artery, and arterial size at the access site were noted. Intra‐ and postprocedural complications, including conversion to a femoral approach, were also recorded. Technical success was defined as successful cannulation of the radial artery followed by catheterization of the target vessels.

### Radial Artery Catheterization Procedure

The patients were identified, brought to the angiography suite, and placed supine on the X‐ray fluoroscopy table. All pressure points were padded. The patients were draped under sterile conditions and sedated using either general anesthesia or moderate conscious sedation, depending on the individual patient's medical status. In all cases, the right radial region was selected for access. A 21‐gauge needle was used to infiltrate the wrist with a local anesthetic (2–3 mL lidocaine 2%), and a modified Seldinger technique was used to gain access. A combination of ultrasound guidance and manual palpation was used to locate the proximal radial artery. A “radial cocktail” including intra‐arterial verapamil (2.5 mg), nitroglycerin (200 μg in 0.5 mL saline), and heparin was injected to prevent vasospasm. A 5‐F slender radial artery sheath with a 5F, 95‐ to 100‐cm Simmons 2 diagnostic catheter (Merit Medical, Salt Lake City, UT) was used for performing the diagnostic procedures; 6F versions of the sheath and catheter were used for the interventional procedures. A 0.035‐inch guidewire was used to gain access intracranially in all procedures. Heparin was given to all patients at an initial dose of 50 to 60 IU/kg of body weight. An additional dose was given on the basis of activated clotting time. In all cases, heparin was administered intra‐arterially via the radial sheath. Following placement of the sheath, selective catheterization of the target blood vessels, as required in the procedure, was performed. After obtaining appropriate angiographic images, all wires and catheters were removed. A completion run was performed to look for any intraluminal thrombus, extravasation, dissection flap, or residual/persistent vasospasm in the radial artery. The sheath was removed and a vascular band or a compressive dressing was applied for closure. The patients were monitored for any complications after the procedure, with special note of bleeding or hematoma formation at the access site.

### Literature Review

Systematic searches of MEDLINE (National Library of Medicine), PubMed (National Library of Medicine), and Embase (Elsevier) databases were conducted using Preferred Reporting Items for Systematic Reviews and Meta‐Analyses guidelines. Keywords with Boolean operators (“radial artery” AND “pediatric”) were used to increase specificity and sensitivity. Screening and initial study selection for the systematic review was conducted using Rayyan (Rayyan Systems Inc., Cambridge, MA), a web‐based application for meta‐analysis and systematic review. We included all articles published in English between January 2000 and September 2021 and reporting ≥4 pediatric patients in whom the TRA was used to perform either diagnostic or interventional procedures. Conference abstracts, background articles, cadaveric and animal studies, and systematic reviews and meta‐analyses were excluded. Studies in which data specific to the TRA in the pediatric population could not be separately extracted or those in which diagnostic and intervention cohorts were separately reported with overlap were also excluded. Full‐text articles were assessed regarding the time period of the study, and those with overlapping patient populations were excluded while maintaining the most recent and complete study. Data extracted included patient sample size, mean age, mean arterial size at the access site, contrast and fluoroscopy radiation doses, technical success rate, femoral crossover, and rate of radial artery vasospasm.

### Statistical Analysis

Continuous variables were reported as means and respective SDs and medians and interquartile ranges according to data normality. Categorical variables were reported as frequencies. For our systematic review analysis, we included our study's results and estimated rates of success; vasospasm, femoral crossover, and mean arterial diameters were generated, with weighting for each study sample size. The extent of heterogeneity among studies was assessed with an *I* ^2^ test. Pooled analyses were performed with fixed‐effect and DerSimonian and Laird random‐effects models, and the results summarized on forest plots. All analyses were completed using RStudio version 1.4.1106 and the R General Package for Meta‐Analysis (RStudio, Boston, MA).

## Results

### Clinical Characteristics

Twenty‐one patients were included in our study. Mean age was 16.6±2.23 years (range, 9–17 years), and 11 (52.4%) were male sex. Mean height was 65.4 inches and mean weight 166.4 pounds, with a mean body mass index of 23.5. The TRA was used for diagnostic angiography in 15 cases (71.4%), with intervention being done in the remaining 6 (28.6%). The most common indication for diagnostic angiography was intracranial aneurysm in 7 (33.3%), followed by headache secondary to trauma in 3 (14.3%), and arteriovenous malformation in 2 cases (9.5%). These and other characteristics and indications are summarized in Table 1.

**Table 1:** Patient Demographics and Indications for Diagnostic and Interventional Neuroangiography

Demographics	n (%)*
Age, mean y±SD	16.6±2.2
Male sex	11 (52.4)
Height, mean in inches±SD	65.4±3.6
Weight, mean in lb±SD	166.4±52.4
Body mass index, mean±SD	23.5±9.3

**Indications for angiography**	n=21
*Diagnostic*	15 (71.4)
Intracranial aneurysm	7 (33.3)
Headache secondary to trauma	3 (14.3)
Arteriovenous malformation	2 (9.5)
Nasopharyngeal angiofibroma	1 (4.8)
Hyperintensity on magnetic resonance imaging	1 (4.8)
Idiopathic intracranial hypertension	1 (4.8)
*Intervention*	6 (28.6)
Aneurysm embolization	2 (9.5)
Nasopharyngeal angiofibroma	2 (9.5)
Arteriovenous malformation embolization	1 (4.8)
Thrombectomy for stroke	1 (4.8)

^*^ Number and percentage of patients unless otherwise indicated.

### Procedural Information and Outcomes

**Figure 1:**
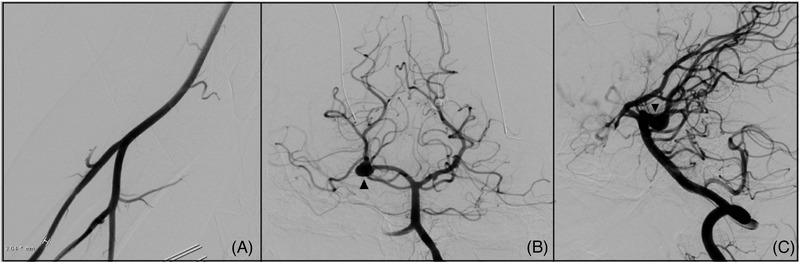
**A case showing normal radial access**. **A**, Anterior‐posterior view demonstrating the radial artery access site with diameter measurement. **B**, Left vertebral artery cranial injection in the anterior‐posterior plane demonstrating a right P1‐P2 junctional aneurysm (indicated by arrowhead). **C**, Lateral view of the aneurysm seen with normal caliber and course of left vertebral artery, and right vertebral arteries (indicated by arrowhead).

Overall, mean procedure time was 62.4±48.9 minutes. Wrist infiltration with lidocaine as local anesthetic was performed in all 21 cases. The right proximal radial artery was the access site in all cases. No distal or left‐sided TRAs were done. Verapamil (2.5 mg) as part of the radial cocktail was administered in all cases (Figure 1, Table 2).

**Table 2:** Procedural Details

Characteristics	n (%)*
Mean radial artery diameter, mm±SD	2.2±0.5
Moderate conscious sedation	13 (61.9)
General anesthesia	8 (38.1)
Vascular band closure	19 (90.5)
Compressive dressing closure	2 (9.5)
Vasospasm rate	2 (9.5)
Conversion to femoral access	2 (9.5)
Postprocedure complications	0 (0)
Total fluoroscopy time, min, mean±SD	18.0±17.2
Total radiation exposure, Gy, mean±SD	1.41
Total contrast dose, Gy, mean±SD	0.52

^*^ Number and percentage unless otherwise indicated.

Moderate conscious sedation was the preferred form of anesthesia in 13 cases (61.9%), and general anesthesia was used in the remaining 8 (38.1%). In one case, a 12‐year‐old patient was initially sedated using moderate conscious sedation but soon after started to display signs of anxiety and restlessness. The patient eventually was sedated with general anesthesia and radial access gained successfully thereafter.

The mean radial artery diameter at the access site was 2.2±0.5 mm. Mean fluoroscopy time was noted to be 18.0±17.2 minutes, with a mean total radiation exposure of 1.41 Gy. A radial artery compression device, TR Band (Terumo Medical Corporation, Somerset, NJ), was used to achieve hemostasis in 19 cases (90.5%). A compressive dressing was used in 2 cases (9.5%).

In terms of procedure complications and outcomes, 2 cases (9.5%) required conversion to femoral access. The first case was an adolescent boy, details of which are presented below in the case illustration. The second instance of complication was seen in a teenage girl referred for diagnostic angiography. The patient was found to have an aberrant right radial artery, and the wire would not advance 2 to 3 cm beyond the puncture site. Therefore, a radial approach was abandoned, and crossover to a femoral approach was undertaken. Two patients (9.5%) suffered vasospasm that was successfully treated with a dose of verapamil (2.5 mg) in addition to the standard radial cocktail. Notably, no postprocedure permanent radial artery occlusions or permanent neurologic deficits were noted in our cohort. Patients were monitored for complications (radial artery occlusion, neurologic deficit, or hematoma formation at the access site) for 30 days after the procedure. Additional data regarding outcomes are shown in Table 3.

**Table 3:** Studies Included in the Systematic Review

Study (y)	Study design	Sample size, no. patients	Mean age, y	Mean fluoroscopy time, min	Mean arterial size, mm	Mean vasospasm, %	Mean success rate, %
Majmundar et al^16^ (2020)	Retrospective	4	14	69.3	2.3	25	100
Srinivasan et al^18^ (2020)	Retrospective	47	14.1	12.3	2.1	13.1	91.8
Alshehri et al^19^ (2021)	Retrospective	20	14.6	33.7	2.6	0	100
Cox et al^17^ (2021)	Retrospective	52	14.9	9.5	…	0	89.4
Current series	Retrospective	21	16.6	23.9	2.2	9.5	90.5

“…” indicates data not reported.

[Correction added on November 8, 2022, after first online publication: The value in the column “Mean vasospasm, %” for the study Cox et al (2017) was changed from … to 0.

### Case Illustration

**Figure 2:**
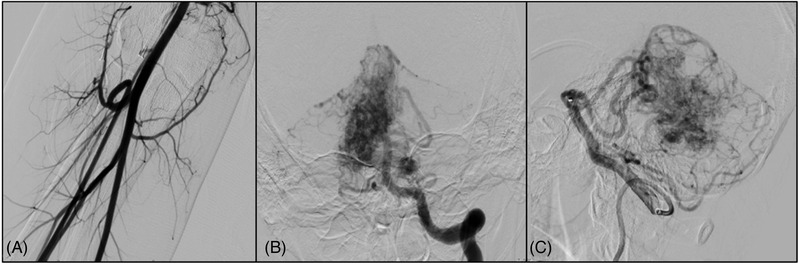
**Case illustration**. **A**, A radial loop demonstrated on anterior–posterior angiographic view. The ulnar and interosseous arteries come off in a straight fashion. After several attempts to cross the loop, the radial artery approach was abandoned, and the procedure was performed transfemorally. **B**, Anterior‐posterior view demonstrating a large arteriovenous malformation in the posterior fossa coming off of the superior cerebellar artery. Also appreciated are multiple feeders to the arteriovenous malformation. **C**, Lateral view demonstrating the arteriovenous malformation.

**Figure 3:**
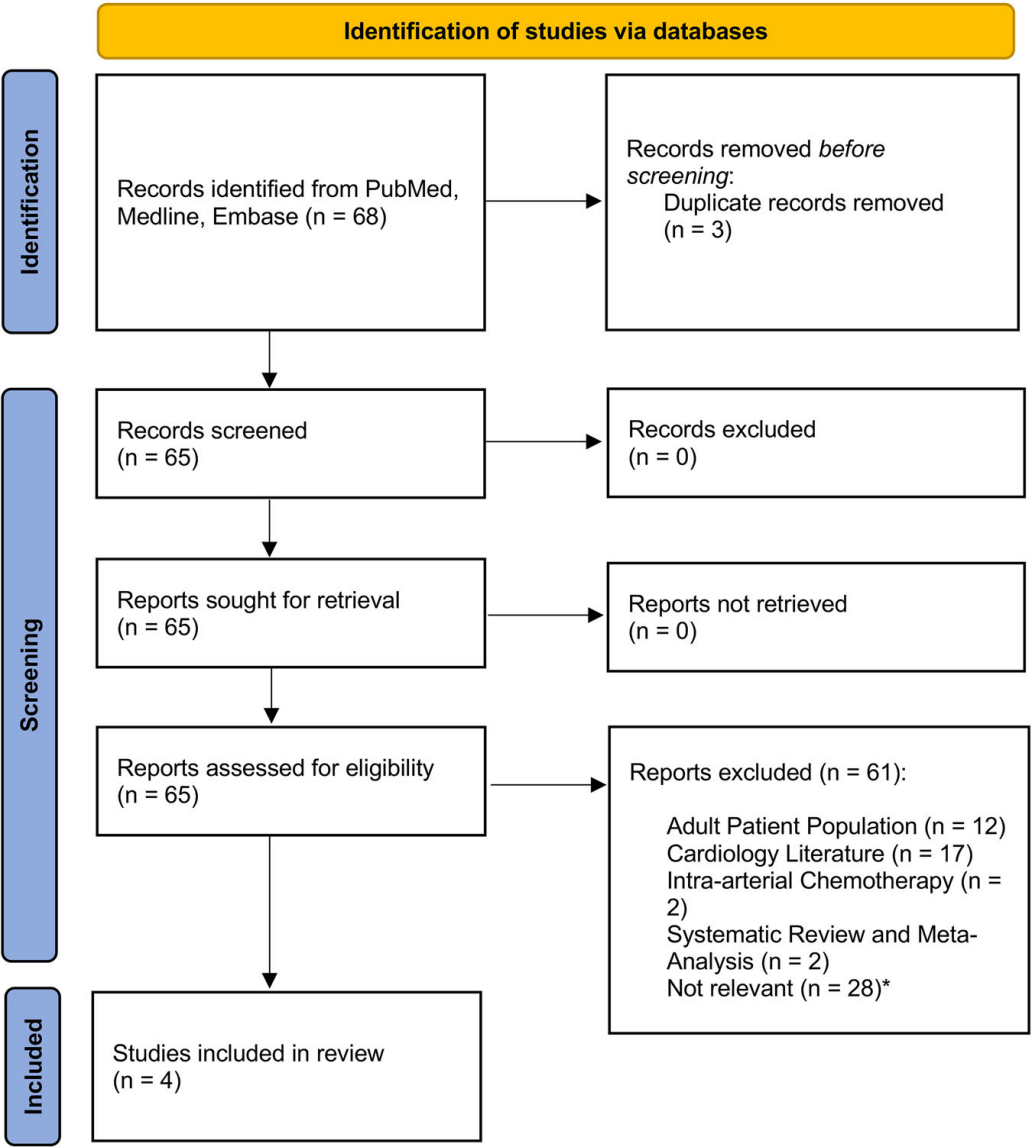
**Preferred Reporting Items for Systematic Reviews and Meta‐Analyses flow chart showing the study identification and selection process**. Template obtained from Page MJ, McKenzie JE, Bossuyt PM, Boutron I, Hoffmann TC, Mulrow CD, et al. The PRISMA 2020 statement: an updated guideline for reporting systematic reviews. BMJ 2021;372:n71. https://doi.org/10.1136/bmj.n71.

An adolescent boy presented with a 3‐day history of headache following a rugby injury. Noncontrast computed tomography revealed an incidental posterior fossa arteriovenous malformation with probable hemorrhage (Figure 2). After the administration of sedatives, the patient's right wrist was infiltrated using a local anesthetic (2–3 mL lidocaine 2%). Under ultrasound guidance, the right radial artery was accessed in the usual sterile fashion, and a 5F sheath was placed. The patient experienced mild wrist pain despite good sheath placement angiographically. A radial loop was encountered just distal to the cubital fossa (Figure 2). [Correction added on November 8, 2022, after first online publication: In the preceding sentence, the figure citation was changed from Figure 3 to Figure 2.] Of note, before traversing the radial loop, verapamil (2.5 mg), heparin (2500 international unit), and nitroglycerin (200 μg in 0.5 mL saline) were administered as part of the routine “radial cocktail.” Several attempts were made to straighten the radial loop and traverse it; however, after each try, the radial artery would straighten out temporarily but then rekink, causing inability to complete the angiogram. Therefore, we aborted the radial approach, closed using a vascular band, and proceeded with a femoral approach. The procedure was thereafter completed transfemorally with no other complications, and the patient was discharged the same day.

### Systematic Review of the Literature

**Figure 4:**
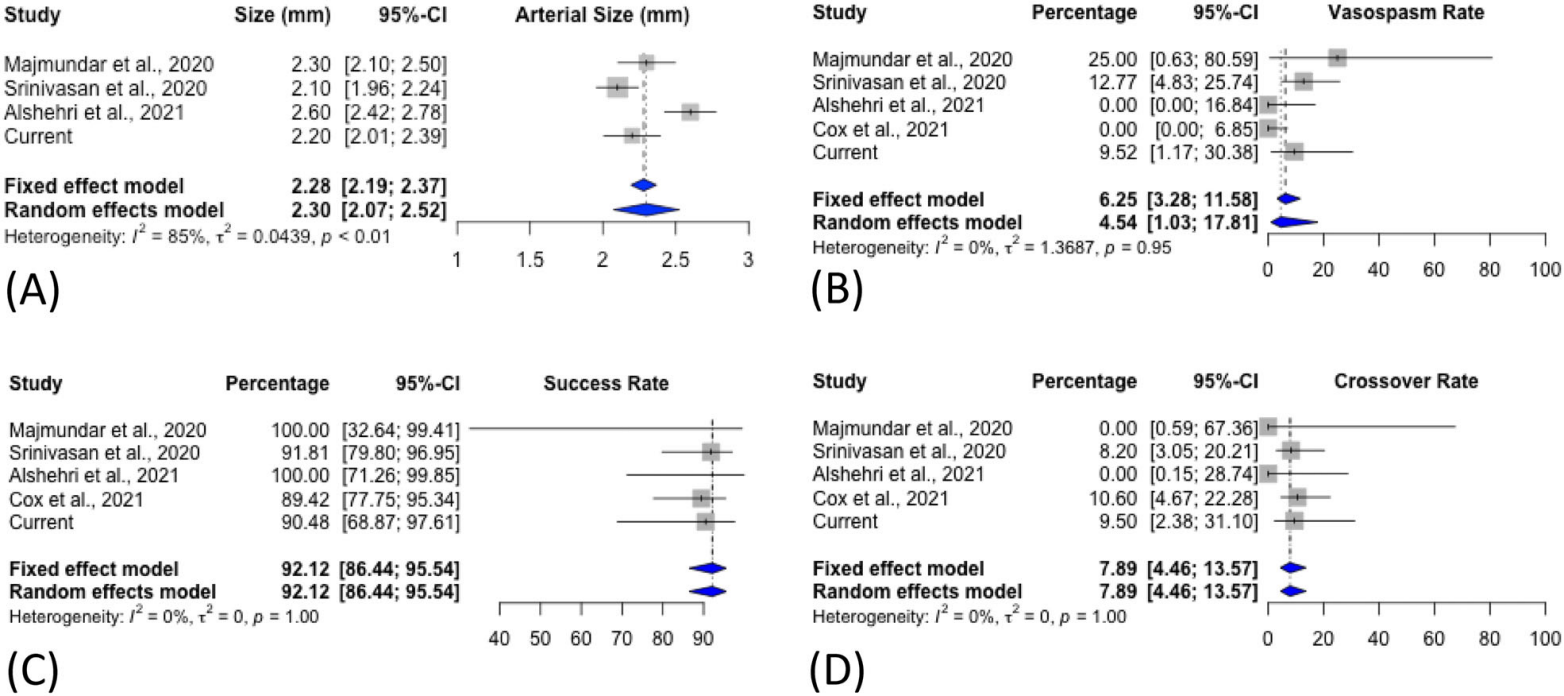
**Systematic Review Results**. Forest plots showing arterial size (mm) (**A**), vasospasm (percentage of cases) (**B**), success (**C**), and crossover rates (percentages of cases) (**D**) for studies included in the systematic review. Arterial size was not reported in the study by Cox et al.^17^ [Correction added on November 8, 2022, after first online publication: The image for Figure 4 was changed in this version to correct part B.]

Our search resulted in 68 articles, of which 3 were duplicates. From 65 unique articles, 61 were excluded for the reasons listed in Figure 3. [Correction added on November 8, 2022, after first online publication: In the preceding sentence, the figure citation was changed from Figure 4 to Figure 3.] The remaining 4 studies were assessed in full text, and all 4 fulfilled the inclusion criteria. A total of 4 articles plus our cohort were included in the final analysis for a total of 144 pediatric patients (Table 3). The estimated rate of procedural success was 92% (95% CI, 86%–96%), [Correction added on November 8, 2022, after first online publication: The preceding values have been changed from “93% (95% CI, 74%–113%)” to “92% (95% CI, 86%–96%)” in this version.] with significant vasospasm seen in 6.3% (95% CI, 3%–12%). [Correction added on November 8, 2022, after first online publication: The preceding values have been changed from “7% (95% CI, 1%–12%)” to “6.3% (95% CI, 3%–12%)” in this version.] These rates are plotted in Figure 4. Pooling of the data revealed a mean age of 14.8 years and a mean access‐site arterial diameter of 2.2 mm.

## Discussion

In this study, we report our clinical experience and present a systematic review of the literature on the use, safety, and effectiveness of radial access in pediatric patients undergoing diagnostic and interventional neuroangiography. We obtained a high technical success rate (90.5%) and a low rate of reversible arterial vasospasm (9.5%), with an acceptable incidence of femoral crossover (9.5%). Our systematic review showed similar results in terms of vasospasm rates and procedural outcomes.

### Rationale for Pediatric Population

The TFA has been a standard of practice in pediatric neurointervention for the past 2 decades, with the TRA being practiced very recently.^16^, ^17^, ^18^, ^19^ Encouraging results from several small and large multicenter endovascular experiences with the TRA in adults have paved the way to explore radial access in exclusively pediatric populations.^8^, ^9^, ^11^, ^12^, ^13^, ^14^, ^15^, ^21^ Exploring radial access in pediatric patients is especially important, as femoral site complications, such as acute vessel occlusion, thromboses, and loss of arterial pulse, although rare in adults, are a major concern among pediatric patients as reported in the cardiology experience.^22^, ^23^, ^24^, ^25^ Alexander et al^22^ evaluated risk factors for acute loss of arterial pulse in children <18 years of age undergoing cardiac catheterizations through the femoral artery and reported an overall rate of 4.7%. For children <6 months of age, the incidence of loss of arterial pulse requiring treatment was 13.6%; a significantly higher rate than that for the rest of the cohort.^22^ Limb length shortening secondary to growth plate arrest, although not a concern in adults, has been reported for neonates who require femoral arterial line access. This is particularly important for preterm neonates who require longer hospital stays. In comparison, the reported incidence of thrombotic complications from intra‐arterial catheter placement in the femoral artery in adults ranges from 0.18% to 1.45%. Similarly, Kamyszek et al^26^ investigated the incidence of femoral artery thromboses in infants (0–12 months of age) undergoing cardiac catheterizations. Of a total of 509 catheterizations, 40 cases (7.9%) of femoral artery thromboses were documented.^26^ These results point to a significantly higher incidence of thromboses and complication rates associated with loss of arterial pulse in pediatric populations when compared with adults. It is important to acknowledge the potential bias in the literature discussed above, as a number of studies have reported outcomes and complication rates for patients <2 years of age in whom transradial access is more technically challenging.

Increased vessel diameter of the femoral arteries in adults has also been linked to a lower frequency of intra‐arterial catheter‐related thrombosis.^27^ The incidence of arterial closure device failure is higher in children undergoing femoral catheterization than in adults.^28^, ^29^, ^30^ This may be attributable to the lack of smaller closure devices tailored to pediatric femoral vessel sizes. Thus, longer immobilization periods and manual pressure are often required in these patients, which can be problematic, especially in pediatric populations with neurologic disorders. This may also require sedation using general anesthesia and hamper early mobilization.^18^

### Special Considerations

Although the TRA in the pediatric population has high technical success rates as shown by our review (fixed‐effect model rate, 0.92; 95% CI, 0.86–0.96), [Correction added on November 8, 2022, after first online publication: The preceding values have been changed from “0.93; 95% CI, 0.74–1.13” to “0.92; 95% CI, 0.86–0.96” in this version.] special considerations are required in pediatric populations concerning the challenges associated with vasospasm, small vessel size, and acute radial artery occlusion in these patients.

### Radial Artery Vasospasm

One of the most frequently encountered challenges in radial access is radial artery vasospasm. The radial artery is more vasoreactive and vulnerable to spasm after local trauma because of the abundance of α‐adrenoreceptors in the adventitia.^31^ Several studies in adult patients have demonstrated ways to reduce the incidence of potential vasospasm using a radial cocktail of vasodilators and spasmolytics administered before commencing the procedure.^32^, ^33^ At our institute, we have implemented this practice in both adult and pediatric patients. In our review, all studies reporting the TRA in pediatric patients reported using some combination of a radial cocktail consisting of verapamil, heparin, and nitroglycerin.^16^, ^17^, ^18^, ^19^ Rates of vasospasm ranged from a high of 25% to a low of 0% (fixed‐effect model rate, 0.06; 95% CI, 0.03–0.12). [Correction added on November 8, 2022, after first online publication: The preceding values have been changed from “0.07; 95% CI, 0.01–0.12” to “0.06; 95% CI, 0.03–0.12)” in this version.] This unusually high vasospasm rate of 25% can be explained by the fact that only 4 patients were included in the study by Majmundar et al,^16^ of which 1 suffered mild vasospasm that resolved on administration of intra‐arterial verapamil. In our cohort, only 2 patients (9.5%) suffered mild vasospasm that was resolved with administration of 2.5 mg of verapamil. These rates are in line with those reported for adult patients undergoing a TRA.^12^ A recent meta‐analysis performed on 17 comparative studies of complications of a TRA versus a TFA for neuroendovascular procedures demonstrated that access‐site complications were significantly lower in a TRA (1.8% versus 3.2% for TRF; *P*<0.001).^9^ The TRA was also associated with lower odds of access‐site complications than TFA (odds ratio, 0.42; 95% CI, 0.25–0.68; *P*<0.001; *I* ^2^=31%).^9^ It is important to note that in their meta‐analysis, Schartz et al^9^ reported access site complications including wrist hematomas, site abscesses, radial artery occlusions (both symptomatic and asymptomatic), significant access‐site pain, pseudoaneurysms, and symptomatic vasospasms without giving a breakdown of the rates of each of these complications, which makes it difficult to make a fair judgment regarding individual rates. Vasospasm rates for adult patients fall in the range of 4% to 20%.^34^, ^35^ Routine prophylactic use of vasodilators and antispasmodics can help prevent this known and reversible complication.^35^ In cases in which arterial vasospasm is encountered midprocedure, the first step should be to administer higher doses of antispasmodics (verapamil or nitroglycerin) through the guide catheter or the sheath.^32^ In cases of severe vasospasm where catheter movement is restricted, it is advisable to place the patient under general anesthesia, as increased sedation has shown to reduce the incidence of radial artery vasospasm.^32^ This is already practiced in pediatric patients because most procedures are carried out under general anesthesia. This was demonstrated aptly by Alshehri et al,^19^ who reported no incidence of radial artery vasospasm following the use of general anesthesia or anxiolytics in their cohort. In our systematic review, we were also able to identify reasons for an unsuccessful TRA and eventual crossover to a TFA, which included severe radial artery vasospasm causing limited access and subsequent catheter movement as well as inability to catheterize the radial artery because of limited ultrasound guidance usage. We demonstrate that most, if not all, of these complications may be avoided with routine use of a “radial cocktail” and ultrasound guidance.

### Radial Artery Size, Catheter Use, and Fluoroscopy in Children

Difficulty accessing and navigating the smaller‐diameter radial arteries in pediatric patients can be best addressed with routine use of ultrasound guidance and selection of appropriately sized catheters. In adult patients, a 5F slender sheath (2.13 mm outer diameter, 1.78–1.9 mm inner diameter) is used for routine diagnostic angiography through the radial artery because these sheaths are associated with lower radial artery occlusion rates and procedural failure.^36^ In pediatric patients, despite smaller‐sized vessels, essentially the same setup is used. Interestingly, our review revealed that all studies except 1 reported using either a 5F or a 6F sheath.^16^, ^17^, ^18^, ^19^ For routine diagnostic angiography, most studies reported using the 5F slender sheath and for interventions, such as arteriovenous malformation and aneurysm embolization, 6F was preferred; the same trend was followed in our cohort.^16^, ^17^, ^18^, ^19^ Although no consensus exists regarding the minimum radial artery diameter feasible for access, ≤1 mm is generally considered the lower limit, especially for younger patients (≤4 years of age).^16^, ^17^, ^18^, ^19^ Srinivasan et al reported a cutoff of 1.4 mm, although the decision was left to individual interventionists.^18^ The pooled mean radial artery diameter in our review was 2.2 mm, which is on par with that reported in the adult literature.^36^ In addition, the use of long sheaths has shown promise in preventing radial artery spasms and occlusions.^37^, ^38^ Long sheaths cover a greater length, spanning from puncture site to the larger brachial artery, and can provide additional protection from manipulation of catheters during the procedure.^37^, ^38^

Fluoroscopy times and dosage are also an important consideration in the pediatric population. Our review revealed a pooled mean fluoroscopy time of 22.1 minutes for both diagnostic and interventional procedures. There is a dearth of literature for comparative results in terms of fluoroscopy times and outcomes. Alshehri et al^19^ compared procedural outcomes and fluoroscopic parameters between the TRA and TFA for pediatric patients and reported no significant difference in terms of mean fluoroscopy duration (33.7±0.2 minutes versus 23.3±26.2 minutes; *P*=0.34) and mean fluoroscopy dose received by each group (150.9±133.7 and 122.9±79.7 Gy cm^2^; *P*=0.43), respectively. In our cohort, the mean fluoroscopy time was 18.0±17.2 minutes, which is comparable to the transfemoral group reported by Alshehri et al We hypothesize that the difference between these times may increase as more operators gain experience in using the TRA in smaller vessels. This is important because minimizing fluoroscopy time and dose is especially critical in pediatric patients as compared with their adult counterparts.

### Limitations

The foremost limitation of our study is the retrospective and observational nature of the patient data in our cohort. The systematic review also contains retrospective case studies that looked at small populations and may have been influenced by publication bias. However, we present some key findings in terms of patient selection and technical details of the TRA in pediatric patients.

## Conclusion

This case series and literature review suggest that pediatric neurointervention performed through the radial artery is feasible and effective. Routine use of ultrasound guidance, use of long sheaths, selection of appropriately sized catheters, and prophylactic use of vasodilators and antispasmodics can help with the success of the procedure and limit common access‐site complications.

## Author Contributions

Conception and design, drafting the manuscript: Ammad A Baig. Acquisition of the data: Ammad A Baig, Jenna Neumaier, and Yusuf J Hashmi. Analysis and interpretation of the data, critically revising the manuscript, and reviewed submitted version of manuscript: all authors.

## Sources of Funding

This research received no specific grant from any funding agency in the public, commercial, or not‐for‐profit sector.

## Disclosures

Ammad A. Baig, Jenna Neumaier, Yusuf J. Hashmi, Muhammad Waqas, Justin M. Cappuzzo, Andre Monteiro, Hamid H. Rai, Wasiq Khawar, and Renee M. Reynolds: None. Jason M. Davies reports the following: research grants: National Institutes of Health National Institute of Neurological Disorders and Stroke, National Science Foundation Small Business Innovation Research; consulting fees: Medtronic; honoraria: Medtronic; support for attending meetings or travel: Medtronic; patents planned, issued, or pending: QAS.ai; participation on a data safety monitoring board or advisory board: National Institutes of Health National Institute of Neurological Disorders and Stroke Strokenet; stock or stock options: Synchron, Cerebrotech, QAS.ai, and RIST. Kenneth V. Snyder reports the following: consulting fees: Boston Scientific, Canon Medical Systems USA, Inc., MicroVention, Medtronic, and Stryker Neurovascular; payment or honoraria for lectures, presentations, speakers’ bureaus, manuscript writing, or educational event: Canon Medical Systems USA Inc; stock or stock options: Boston Scientific, Access Closure Inc, and Niagara Gorge Medical. Elad I. Levy reports the following: consulting fees: Claret Medical, GLG Consulting, Guidepoint Global, Imperial Care, Medtronic, Rebound, StimMed, Misionix, Mosiac, Clarion, and IRRAS; payment or honoraria for lectures, presentations, speakers bureaus, manuscript writing, or educational events: Medtronic; payment for expert testimony: for rendering medical/legal opinions as an expert; support for attending meetings or travel: reimbursement for travel and food for some meetings with the Congress of Neurological Surgeons and American Board of Neurological Surgery; stock or stock options: NeXtGen Biologics, RAPID Medical, Claret Medical, Cognition Medical, Imperative Care, Rebound Therapeutics, StimMed, and Three Rivers Medical. Adnan H Siddiqui reports the following: consulting fees: Amnis Therapeutics, Apellis Pharmaceuticals, Inc., Boston Scientific, Canon Medical Systems USA, Inc., Cardinal Health 200, LLC, Cerebrotech Medical Systems, Inc., Cerenovus, Cerevatech Medical, Inc., Cordis, Corindus, Inc., Endostream Medical, Ltd, Imperative Care, Integra, IRRAS AB, Medtronic, MicroVention, Minnetronix Neuro, Inc., Penumbra, Q'Apel Medical, Inc., Rapid Medical, Serenity Medical, Inc., Silk Road Medical, StimMed, LLC, Stryker Neurovascular, Three Rivers Medical, Inc., VasSol, Viz.ai, Inc., and W.L. Gore & Associates; leadership or fiduciary role in other board, society, committee, or advocacy group: past secretary of the Board of the Society of NeuroInterventional Surgery, chair of the Cerebrovascular Section of the American Association of Neurological Surgeons/Congress of Neurological Surgeons; stock or stock options: Adona Medical, Inc., Amnis Therapeutics, Bend IT Technologies, Ltd., BlinkTBI, Inc, Buffalo Technology Partners, Inc., Cardinal Consultants, LLC, Cerebrotech Medical Systems, Inc, Cerevatech Medical, Inc., Cognition Medical, CVAID Ltd., E8, Inc., Endostream Medical, Ltd, Imperative Care, Inc., Instylla, Inc., International Medical Distribution Partners, Launch NY, Inc., NeuroRadial Technologies, Inc., Neurotechnology Investors, Neurovascular Diagnostics, Inc., PerFlow Medical, Ltd., Q'Apel Medical, Inc., QAS.ai, Inc., Radical Catheter Technologies, Inc., Rebound Therapeutics Corp. (Purchased 2019 by Integra Lifesciences, Corp), Rist Neurovascular, Inc. (Purchased 2020 by Medtronic), Sense Diagnostics, Inc., Serenity Medical, Inc., Silk Road Medical, SongBird Therapy, Spinnaker Medical, Inc., StimMed, LLC, Synchron, Inc., Three Rivers Medical, Inc., Truvic Medical, Inc., Tulavi Therapeutics, Inc., Vastrax, LLC, VICIS, Inc., Viseon, Inc.; other financial or nonfinancial interests: national PI/steering committees: Cerenovus EXCELLENT (Revascularization for Acute Ischemic Stroke with Embotrap presenting within 8 hours of onset and large vessel occlusion) and ARISE II (Trial Analysis of Revascularization in Ischemic Stroke With EmboTrap) trial; Medtronic SWIFT PRIME (Medtronic SWIFT PRIME Solitaire FR With the Intention For Thrombectomy as Primary Endovascular Treatment for Acute Ischemic Stroke [SWIFT PRIME] Clinical Trial), VANTAGE (A Study of the Pipeline Vantage Embolization Device With Shield Technology™ for Endovascular Treatment of Wide‐Necked Intracranial Aneurysms), EMBOLISE (A Study of the Embolization of the Middle Meningeal Artery With ONYX Liquid Embolic System In the Treatment of Subacute and Chronic Subdural Hematoma) and SWIFT DIRECT trials (Solitaire With the Intention For Thrombectomy Plus Intravenous t‐PA Versus DIRECT Solitaire Stent‐Retriever Thrombectomy in Acute Anterior Circulation Stroke); MicroVention FRED Trial (Pivotal Study of the MicroVention Flow Re‐Direction Endoluminal Device [FRED] Stent System in the Treatment of Intracranial Aneurysms) & CONFIDENCE Study (Pivotal Study of the MicroVention, Inc. Carotid Artery Stent System Used in Conjunction With the Nanoparasol Embolic Protection System for the Treatment of Carotid Artery Stenosis in Patients at Elevated Risk for Adverse Events From Carotid Endarterectomy); MUSC POSITIVE (Perfusion imaging Selection of Ischemic Stroke Subjects for Endovascular Therapy) trial; Penumbra 3D Separator Trial (A randomized, concurrent controlled trial to assess the safety and effectiveness of the Separator 3D as a component of the Penumbra System in the revascularization of large vessel occlusion in acute ischemic stroke), COMPASS Trial (a Direct Aspiration First Pass Technique), INVEST (A Single Arm, Feasibility Study of Minimally Invasive Endoscopic Surgical Treatment with Apollo for Supratentorial Intracerebral Hemorrhage [ICH]) trial, MIVI neuroscience EVAQ Trial (A Prospective, Multi‐Center, Single Arm Study to Evaluate the Q Revascularization System for Neurointervention in Acute Ischemic Stroke: The EvaQ Study), Rapid Medical SUCCESS Trial (Success in Comaneci‐Assist Coils Embolization Surveillance Study), and InspireMD C‐GUARDIANS IDE pivotal trial (A Multicenter, Single‐Arm, Pivotal Study to Evaluate the Safety and Efficacy of the CGuard™ Carotid Stent System When Used to Treat Symptomatic and Asymptomatic Carotid Artery Stenosis in Patients Undergoing Carotid Artery Stenting).
